# De-Ritis Ratio Is Associated with Mortality after Cardiac Arrest

**DOI:** 10.1155/2020/8826318

**Published:** 2020-11-04

**Authors:** Zhengri Lu, Genshan Ma, Lijuan Chen

**Affiliations:** Department of Cardiology, Zhongda Hospital, School of Medicine, Southeast University, Nanjing, China

## Abstract

**Introduction:**

The aim of our study was to explore the associations of the aspartate transaminase/alanine transaminase (De-Ritis) ratio with outcomes after cardiac arrest (CA).

**Methods:**

This retrospective study included 374 consecutive adult cardiac arrest patients. Information on the study population was obtained from the Dryad Digital Repository. Patients were divided into tertiles based on their De-Ritis ratio. The logistic regression hazard analysis was used to assess the independent relationship between the De-Ritis ratio and mortality. The Kaplan-Meier method and log-rank test were used to estimate the survival of different groups. Receiver operating characteristic (ROC) curve analysis was utilized to compare the prognostic ability of biomarkers. A model combining the De-Ritis ratio was established, and its performance was evaluated using the Akaike information criterion (AIC).

**Results:**

Of the 374 patients who were included in the study, 194 patients (51.9%) died in the intensive care unit (ICU), 213 patients (57.0%) died during hospitalization, and 226 patients (60.4%) had an unfavorable neurologic outcome. Logistic regression analysis including potentially confounding factors showed that the De-Ritis ratio was independently associated with mortality, yielding a more than onefold risk of ICU mortality (OR 1.455; 95% CI 1.088-1.946; *p* = 0.011) and hospital mortality (OR 1.378; 95% CI 1.031-1.842; *p* = 0.030). Discriminatory performance assessed by ROC curves showed an area under the curve of 0.611 (95% CI 0.553-0.668) for ICU mortality and 0.625 (0.567-0.682) for hospital mortality. Further, the likelihood ratio test (LRT) analysis showed that the model combining the De-Ritis ratio had a smaller AIC and higher likelihood ratio *χ*^2^ score than the model without the De-Ritis ratio. The Kaplan-Meier curves showed that the CA patients in the De-Ritis ratio tertile 3 group clearly had a significantly higher incidence of ICU mortality (log − rank = 0.007).

**Conclusion:**

An elevated De-Ritis ratio on admission was significantly associated with ICU mortality and hospital mortality after CA. Assessment of the De-Ritis ratio might help identify groups at high risk for mortality.

## 1. Introduction

Cardiac arrest (CA) is a life-threatening emergency situation with an incidence rate of 1 per 1000 patients. Patients presenting after CA have a low survival rate. In patients who achieve return of spontaneous circulation (ROSC), more than half of them die from circulatory shock [1]. There is also a high risk of neurological deficits, leading to high disability and morbidity. CA patients admitted to the intensive care unit (ICU) are usually unconscious. Therefore, the therapeutic management of these patients is challenging work. In spite of advances in cardiopulmonary resuscitation (CPR) and postresuscitation management, the prognosis after CA remains poor. Accurate outcome prediction, especially in the early stages of CA, will help to plan surveillance and treatment strategies [2]. In recent years, the factors influencing the outcome after CA have always been an important part of the research. Clinical outcomes following CA are affected by various parameters [3]. The individualized risk assessment of the patient's potential risk of death or neurological deficits constitutes a crucial diagnostic approach and may influence therapeutic decisions when providing information to family members of patients. The European Resuscitation Council and the European Society of Intensive Care Medicine recommend using a combination of predictors, which include clinical neurological examination, electrophysiological investigations, serum biomarkers, and neuroimaging. However, current guidelines on how to best prognosticate the outcomes of cardiac arrest provide limited guidance and usually focus only on neurological status [4, 5].

In today's era of personalized medicine, the concept of biomarkers for diagnosis and prognosis has become even more important. It requires prognostic markers that can be easily assessed to facilitate the identification of at-risk patients. In this aspect, aspartate aminotransferase (AST) and alanine transaminase (ALT) are easy to assess and, more importantly, are routinely available [6, 7]. It is considered that ALT mainly mirrors liver-specific dysfunction, while AST has been shown to increase after the death of ischaemic cells in some other tissues, including myocardium, skeletal muscle, kidney, and brain. Some studies have suggested that hepatic dysfunction is a common concomitant symptom of cardiovascular disease (CVD) [8, 9]. Evaluation of serum transaminases can predict morbidity and mortality in patients with CVD [6, 10, 11]. Recent studies have shown that AST and ALT may be used as predictors of prognosis in patients after acute and chronic cardiac events [6, 10–12]. We speculate that AST/ALT as a marker might mirror ischaemic end-organ damage in CA. As AST/ALT may provide more additional information on the risk assessment of patients after CA, we hypothesized that AST/ALT is associated with in-hospital death and neurological outcomes of cardiac arrest survivors. Hence, the study was aimed at exploring the associations of the De-Ritis (aspartate transaminase/alanine transaminase; proposed by Fernando De Ritis in 1957) ratio with outcome after CA [13].

## 2. Materials and Methods

### 2.1. Study Population

The primary data used in the study were acquired from the Dryad Digital Repository. The data are accessible in the Dryad Digital Repository (10.5061/dryad.qv6fp83). All patients were collected from a single centre from January 2007 to December 2015. Full details of the study population have been reported previously [14]. This study was a retrospective observational cohort study of adult CA patients treated at the Department of Intensive Care at Erasme Hospital, Brussels (Belgium). The need for informed consent was waived because of its retrospective nature.

### 2.2. Inclusion Criteria

The study included comatose patients with Glasgow Coma Scale (GCS) < 9 who had experienced in-hospital CA (IHCA) or out-of-hospital CA (OHCA). Patients were excluded if they had (1) missing data on liver function; (2) died within 24 hours of ICU admission; (3) no information on CA and CPR; or (4) lack of information on the neurological outcome at 3 months after CA. According to recent resuscitation guidelines, all patients with sustained return of spontaneous circulation (ROSC) received post-CA care [5, 15]. CA survivors in a coma were treated with 24-hour targeted temperature management (TTM; target body temperature: 32–34°C). The standardized institutional protocol of postresuscitation management has been described previously [16].

### 2.3. Data Collection

The following data on the basic characteristics of the patient was collected: demographics, location of arrest, presence of a witness on collapse, and cardiac arrest characteristics. Sequential Organ Failure Assessment (SOFA) score [17] and Acute Physiology and Chronic Health Assessment (APACHE) II score [18] were used to assess the severity of disease within 24 hours of admission. Blood samples were taken on admission as the first available laboratory data obtained after ROSC and processed according to local laboratory standards. Routine laboratory sampling included serum aspartate (AST, normal ranges: <41 IU/L) and serum alanine (ALT, normal ranges: <37 IU/L) transaminases, lactate dehydrogenase (LDH, normal ranges: <200 IU/L), gamma-glutamyl transpeptidase (GGT, normal ranges: 8-38 IU/L), total bilirubin (TBIL, normal ranges: ≤1.2 mg/dL), prothrombin time (PT, normal ranges: >70%), international normalized ratio (INR, normal ranges: ≤1.2), and platelet (PLT, normal ranges:150-350 × 10^3^/mm [3]). The De-Ritis ratio was calculated as the ratio of AST and ALT. Use of mechanical ventilation, continuous renal replacement therapy (CRRT), intra-aortic balloon pump (IABP), extracorporeal membrane oxygenation (ECMO), vasoactive drugs, and the length of ICU stay was recorded. Comorbidities included chronic heart failure (CHF), hypertension (HTN or HT), coronary artery disease (CAD), diabetes, chronic obstructive pulmonary disease (COPD)/asthma, neurological disease, chronic renal failure (CRF), liver cirrhosis, corticosteroids, and chronic anticoagulation. The use of potentially hepatotoxic drugs/interventions that include paracetamol, amiodarone, *β*-lactams, quinolones, azoles, isoniazid, trimethoprim/sulfamethoxazole (TMP/SMX), metronidazole, and any chemotherapy drugs was documented.

### 2.4. Definitions

Hypertension was defined as a systolic blood pressure (SBP) ≥ 140 mmHg, diastolic blood pressure (DBP) ≥ 90 mmHg, and/or the current use of antihypertensive medications [19]. Diabetes mellitus (DM) was defined as a previous history of diabetes and new hyperglycemia, a fasting serum glucose of ≥7.0 mmol/L (≥126 mg/dL), hemoglobin A1c ≥ 6.2%, or current use of hypoglycemic agents [20]. The definition of acute liver failure (ALF) in this study was based on previously published standards, which included an international normalized ratio (INR) ≥ 1.5 in the presence of encephalopathy [21]. Hypoxic hepatitis (HH) is defined as an increase in serum aspartate transaminase and/or serum alanine transaminase to more than 20-fold the upper normal range without any other cause of hepatic cell necrosis after CA [22]. Acute kidney injury (AKI) was diagnosed according to AKIN criteria by the changes of serum creatinine during hospitalization [23]. Shock was defined as a systolic arterial pressure < 90 mmHg despite adequate fluid resuscitation or use of vasopressor such as dopamine/dobutamine, adrenaline, and others for more than 6 hours.

### 2.5. Outcome Measures

The primary outcome was ICU mortality, which was defined as an all-cause mortality during the ICU stay. The secondary outcomes of study endpoint was a composite of hospital mortality and poor neurological function outcome at 3 months after CA, defined as cerebral performance categories score (CPC) 3-5, at/or around 90 days. And favorable neurological outcome was considered a CPC 1–2. The CPC score ranged from 1 to 5, with 1 for good brain function or mild disability, 2 for moderate disability, 3 for severe disability, 4 for coma or vegetative state, and 5 for brain death. The CPC assessment was performed prospectively during follow-up period or by telephone interview with the general practitioner.

### 2.6. Statistical Analysis

Nonnormally distributed continuous data are shown as medians (25th and 75th percentiles) and compared using the Kruskal-Wallis test. Categorical variables are expressed as counts with percentages and compared using the chi-squared test wherever appropriate. The logistic regression model was used to analyze the influence of De-Ritis ratio on ICU mortality, hospital mortality, and neurological outcome. These variables in the model were selected using a backward and forward stepwise method. The results were presented as an odds ratio (OR) with 95% confidence interval (CI) and an OR per standard deviation (OR per SD) increase. In the multivariate analysis, OR was adjusted for potential confounders, including age, male gender, bystander-witnessed CA, bystander CPR, adrenaline, nonshockable rhythm, chronic heart failure, chronic renal failure, liver cirrhosis, shock, AKI, lowest central venous/mixed venous oxygen saturation (ScvO_2_/SvO_2_) during the ICU stay, lactate, ScvO_2_/SvO_2_, GGT, total bilirubin, and mean arterial pressure (MAP). Kaplan-Meier curves were constructed to visualize differences in survival, and the log-rank test was utilized to declare significance. Receiver operator characteristic (ROC) curves were applied to determine optimal cutoff values of the De-Ritis ratio for mortality, and the predictive validity of the De-Ritis ratio measurements for mortality was assessed using corresponding results for the area under the curve (AUC). In addition, the likelihood ratio test (LRT) was used to calculate values of the goodness of fit between the different multivariate prediction models. In the calculation of the LRT, larger *χ*^2^ values and smaller Akaike information criterion (AIC) values indicate a better model fit. A two-tailed *p* value < 0.05 was defined as statistically significant. Statistical analyses were performed with SPSS Statistics 25 (IBM SPSS, Armonk, New York, NY, USA), STATA 11.0 (STATA-Corp, College Station, Texas, TX, USA), and GraphPad Prism 8® (GraphPad Software, Inc., San Diego, California, USA).

## 3. Results

### 3.1. Distribution of De-Ritis Ratio and Baseline Characteristics

**Figure 1:**
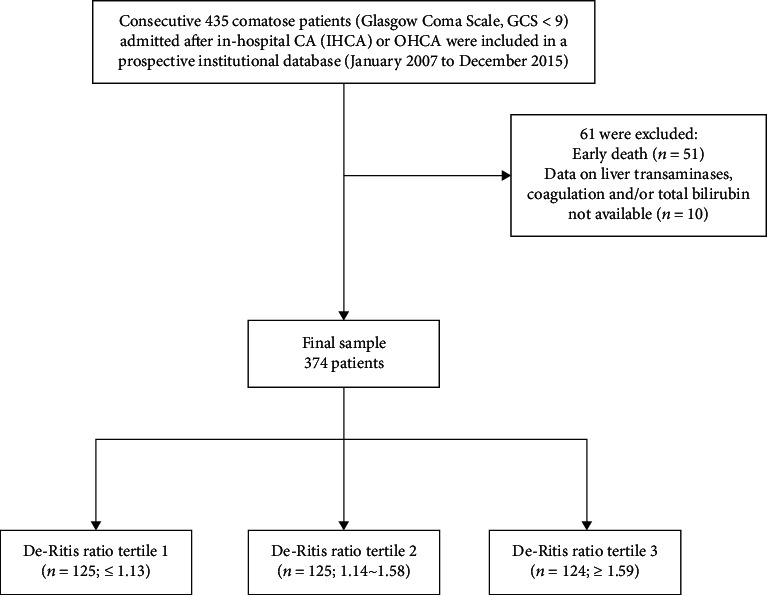
Flowchart of the study population with inclusion and exclusion criteria.

A total of 435 eligible CA patients were admitted, with 61 being excluded. A total of 374 CA patients (270 males, 104 females) were eventually enrolled (Figure 1). The participants were analysed by dividing the De-Ritis ratio into tertiles (tertile 1 group ≤1.13; tertile 2 group 1.14–1.58; and tertile 3 group ≥1.59) and using the first tertile as a reference. Baseline clinical characteristics of the study population at CA are shown in Table 1. Patients in tertile 3 group were older and had a lower rate of OHCA. Significant differences were found among groups in terms of hematology indices on admission, with the exception of ScvO_2_/SvO_2_, proteins, pH, PaCO_2_, PaO_2_, and creatinine. No significant differences were found in the characteristics of comorbidities or comorbidities. The De-Ritis ratio showed a correlation with PT values (*r* = −0.240; *p* < 0.001) and INR values (*r* = 0.226; *p* < 0.001) (Table 2).

### 3.2. Clinical Outcomes

**Figure 2:**
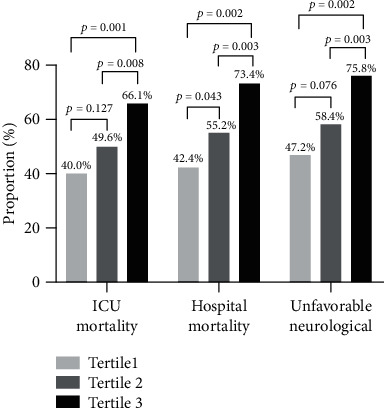
The composite of ICU mortality (primary outcome), hospital mortality, and unfavorable neurological (secondary outcomes) by De-Ritis ratio groups.

Table 3 shows the cumulative incidences of outcomes. Of the 374 patients who were included, 194 patients (51.9%) died in the ICU, 213 patients (57.0%) died during hospitalization, and 226 patients (60.4%) had an unfavorable neurologic outcome. The subjects with a higher De-Ritis ratio at baseline were more likely to have higher ICU mortality, hospital mortality, and unfavorable neurological outcome. For example, ICU mortality (from 40.0%, 49.6%, to 66.1%, *p* < 0.001), hospital mortality (from 42.4%, 55.2%, to 73.4%, *p* < 0.001), and unfavorable neurological outcome (from 47.2%, 58.4%, to 75.8%, *p* < 0.001) increased as the tertile increased (Table 3 and Figure 2). These results suggested that patients with a higher De-Ritis ratio were more inclined to have higher mortality, and this ratio could significantly increase unfavorable neurological outcomes in participants.

We examined the relationship of the De-Ritis ratio (as categorized into tertiles) with ICU mortality, hospital mortality, and unfavorable neurological outcome. In the univariate analysis, the OR for ICU mortality, hospital mortality, and unfavorable neurological outcome significantly increased as the tertiles of the De-Ritis ratio increased. ORs for tertile 3 were significantly higher than the ORs for tertile 1 (unadjusted OR = 2.929; 95% CI: 1.748-4.907, *p* < 0.001 for ICU mortality; unadjusted OR = 3.746; 95% CI: 2.198-6.386, *p* < 0.001 for hospital mortality; unadjusted OR = 3.505; *95% CI*: 2.041-6.018, *p* < 0.001 for unfavorable neurological outcome). Adjustment for the confounding variables did not reduce the ORs for the association between the De-Ritis ratio and ICU mortality (tertile 3 versus tertile 1: adjusted OR = 2.691; 95% CI: 1.443-5.018, *p* = 0.002), hospital mortality (tertile 3 versus tertile 1: adjusted OR = 3.243; 95% CI: 1.716-6.132, *p* < 0.001), and unfavorable neurological outcome (tertile 3 versus tertile 1: adjusted OR = 2.904; 95% CI: 1.532-5.505, *p* = 0.001) (Table 4). Furthermore, we also conducted analyses with the De-Ritis ratio as a continuous variable. We found that, after adjusting for confounders in the multivariate model, the De-Ritis ratio still remained strongly and independently associated with ICU mortality (adjusted OR = 1.455; 95% CI: 1.088-1.946, *p* = 0.011) and hospital mortality (adjusted OR = 1.378; 95% CI: 1.031-1.842, *p* = 0.030) when evaluating the De-Ritis ratio as a continuous variable. However, in the multivariable model, the De-Ritis ratio was not independently associated with unfavorable 3-month neurological outcome. In addition, we observed no effect of AST and ALT (Table 5).

### 3.3. Prognostic Performance, Sensitivity, and Specificity of the De-Ritis Ratio

**Figure 3:**
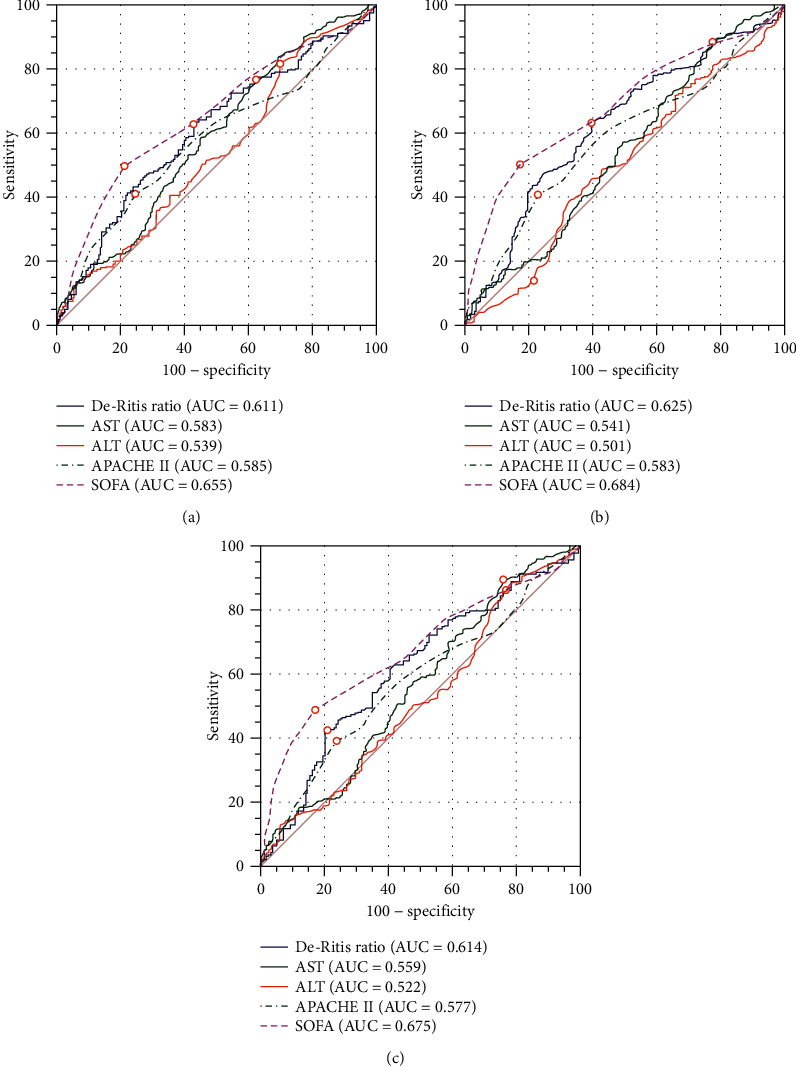
Comparison of ROC curves for predicting ICU mortality, hospital mortality, and unfavorable neurological outcome. (a) The optimal De-Ritis ratio level cutoff point for CA patients with ICU mortality was 1.27 with a sensitivity of 62.9% and a specificity of 57.2%. (b) The optimal De-Ritis ratio level cut-off point for CA patients with hospital mortality was 1.27 with a sensitivity of 63.4% and a specificity of 60.2%. (c) The optimal De-Ritis ratio level cutoff point for CA patients with unfavorable neurological outcome at 3 months was 1.54 with a sensitivity of 42.5% and a specificity of 79.1%.

Discriminatory performance assessed by ROC curves showed an AUC of 0.611 (0.553-0.668) for ICU mortality, 0.625 (0.567-0.682) for hospital mortality, and 0.614 (0.556-0.673) for unfavorable neurological outcome (Figure 3). The optimal De-Ritis ratio cutoff values for CA patients for ICU mortality, hospital mortality, and unfavorable neurological outcome were 1.27, with a sensitivity of 62.9% and specificity of 57.2%; 1.27, with a sensitivity of 63.4% and specificity of 60.2%; and 1.54, with a sensitivity of 42.5% and specificity of 79.1%, respectively.

**Figure 4:**
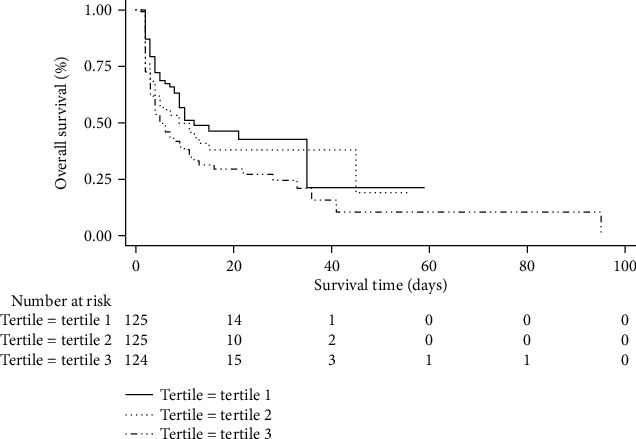
Kaplan-Meier curves for ICU death. Kaplan-Meier curves show significant difference in ICU mortality among the De-Ritis ratio groups (log-rank test, *p* = 0.007). ICU: intensive care unit.

The Kaplan-Meier curves (Figure 4) showed that the CA patients in the De-Ritis ratio tertile 3 group clearly had a significantly higher incidence of ICU death (log-rank *p* = 0.007).

### 3.4. Adding the De-Ritis Ratio to Clinical Information

The comparisons of multivariate model 1 (clinical information: age, sex, adrenaline, bystander CPR (yes/no), and serum lactate level at admission) and multivariate model 2 (age, sex, adrenaline, bystander CPR (yes/no), and serum lactate level at admission and the De-Ritis ratio) were assessed by the AIC and likelihood ratio *χ*^2^ score (Table 6). The AIC values in the multivariate model 1 and model 2 were 476.37 and 463.50, respectively, for ICU mortality. The LRT showed that the *χ*^2^ value of model 2 was larger than that of model 1, and the opposite trend was observed in the AIC value, suggesting that combining the De-Ritis ratio with the multivariate model enabled a superior prediction model for outcomes. Model 1 had moderate performance for ICU mortality, hospital mortality, and poor neurological outcome (AUROC of 0.715, 0.714, and 0.700, respectively). When the De-Ritis ratio was added, the AUROC increased to 0.738, 0.752, and 0.736, respectively.

## 4. Discussion

Our results indicate that a higher De-Ritis ratio was independently associated with higher mortality after CA. As far as we know, the present study is the first that has analysed the impact of the De-Ritis ratio in adult survivors of CA. We demonstrated that patients with a high De-Ritis ratio had a significantly higher association with ICU mortality and hospital mortality than those patients with a low De-Ritis ratio after treatment with TTM, and the results persisted after adjusting for covariates at the patient level. Our study has several strengths. First, we evaluated the neurologic outcome at 3 months after CA rather than at the time of discharge. Second, our study assessed a mixed population including both IHCA and OHCA patients, which may increase the generalizability of the findings, as we think that the prognostic indicators after CA should not be affected by the location of CA.

A previous prospective cohort study showed that a high De-Ritis ratio increased the risk of developing CVD [24]. Yokoyama et al. investigated 3494 Japanese subjects who participated in a community-based health check-up and found that a high De-Ritis ratio was an independent predictor of cardiovascular mortality in a general population [25]. Other studies have also demonstrated similar results, showing that an elevated De-Ritis ratio was significantly associated with an increased risk of CVD mortality [26, 27]. However, these relationships between a high De-Ritis ratio and cardiovascular events have not been reported in patients with CA. The present study found a significant difference in the mortality of CA patients among different De-Ritis ratios.

The De-Ritis ratio is mainly used to judge the severity of chronic liver disease (CLD) [28–30]. In addition, it can also predict the risk of death [31]. However, the underlying mechanism is not entirely understood. The De-Ritis ratio, as an indicator of severity of liver function impairment, may aggregate patients who are more complicated and have a higher probability of death [26]. The manifestation of liver injury usually occurs later than CA. Champigneulle et al. reported that 11% of resuscitated patients after OHCA develop HH [32], and HH was associated with the risk of ICU mortality in multivariate analysis after adjusting for many confounding factors (OR 4.39; 95% CI (1.71, 11.26); *p* < 0.01) [32]. Iesu and his coworkers reported that HH and ALF accounted for 7% and 56%, respectively, of the patients resuscitated after CA [14], and the development of HH rather than ALF was a significant risk factor for poor neurological outcome and ICU mortality [14]. Serum transaminases, such as ALT and AST, are increased when hepatocytes are injured. Therefore, they are used as markers for cytolysis and resolution of liver injury. The elevation of serum transaminases is related to the aetiology and severity of liver injury. Extreme elevations of AST are most often caused by hypoxic hepatitis [33], which is associated with increased mortality [34, 35]. Other similar studies in patients with significantly elevated transaminases have also reported variable aetiologies, mainly including ischaemic-reperfusion injury, extrahepatic cholestasis, and sepsis [36, 37]. It is well-established that severe ischaemia-reperfusion injury after successful resuscitation for CA often leads to multiple organ system injury. Considering the findings of previous studies, the increase of liver enzymes has been attributed to liver injury caused by prolonged ischaemia and ischaemia-reperfusion. This may not explain the increased AST levels and the concomitant lower liver-specific ALT levels. The increase in the De-Ritis ratio may be partly due to nonhepatogenic causes [7]. Serum transaminases should not simply be seen as biomarkers of potential liver injury. They are also key participants in a more systemic phenomenon, such as metabolic syndrome, ischaemia-reperfusion injury, and increased oxidative stress, because all of these are risk factors for death [27, 38, 39]. Considering these findings, the De-Ritis ratio may potentially reflect the systemic hypoperfusion caused by CA.

AST is normally present in both the cytoplasm and mitochondria of hepatocytes, while ALT is mainly distributed in the cytoplasm of hepatocytes [40]. It can be speculated that the De-Ritis ratio may reflect the mitochondrial dysfunction induced by oxidative stress to some extent [26]. In addition, an earlier study of 455 70-year-old ambulatory individuals followed up for 12 years found that low ALT activity was a strong and independent marker of mortality in older people [41]. In our study, patients with a higher De-Ritis ratio were older. The decrease of serum ALT leads to the increase of the De-Ritis ratio, which could reflect hepatic ageing. In addition, liver ageing is associated with increased free radicals, leading to more severe oxidative stress. Our research shows that the De-Ritis ratio is an independent risk factor for death. This means that it can provide more prognostic information about its various components, and the utility the De-Ritis ratio in CVD risk may help with future guideline development. Compared with other existing diagnostic tools, such as the electroencephalogram (EEG) and clinical examination (i.e., pupillary and corneal reactivity and motor responses), the advantages of the De-Ritis ratio include quantitative results and independence from the role of sedatives and/or neuromuscular blocking drugs. In addition, the De-Ritis ratio can be easily obtained and evaluated quickly, and it may provide prognostic information earlier than other existing diagnostic tools. These routine methods of prognostication provide later prognostic information during the initial course of treatment for CA (greater than 72 hours after CA). Furthermore, the US Preventive Services Task Force [42], American Heart Association/American College of Cardiology [43], and Joint British Societies [44] have recommended that the value of novel markers in risk prediction tools can be reconsidered as stronger evidence emerges. We established a multivariable model, including age, sex, adrenaline, bystander CPR (yes/no), and serum lactate level at admission. This clinical information was associated with poor outcome after cardiac arrest. And the serum lactate level is one of the initial laboratory findings that are associated with hospital mortality in patients with various critical illnesses, including CA [45, 46]. In our population, the overall prognostic effectiveness of the De-Ritis ratio for ICU mortality, hospital mortality, and unfavorable neurological outcome was poor (AUC value of 0.611, 0.625, and 0.614, respectively) and was slightly increased when combined with clinical information (AUC value of 0.738, 0.752, and 0.736, respectively). Importantly, adding the De-Ritis ratio to clinical information does indeed improve model performance. Therefore, great care must be taken when applying such a threshold directly to clinical practice.

Another result of this study is that the De-Ritis ratio was associated with neurologic outcomes in CA patients by evaluating it as a categorical variable (tertile 3 versus tertile 1: adjusted OR = 2.904; 95% CI: 1.532-5.505, *p* = 0.001). However, this relationship did not exist after evaluating the De-Ritis ratio as a continuous variable (adjusted OR = 1.281; 95% CI: 0.940-1.745, *p* = 0.117). Campos et al. have confirmed the neuroprotective effect of AST through animal experiments and clinical studies [47]. In rat animal models, they found that the activation of AST reduced the area of cerebral infarction, volume of oedema, and incidence of sensorimotor dysfunction [47]. In patients with AIS, the levels of AST and ALT were negatively correlated with infarct size, and patients with decreased AST were more likely to have early neurological impairment [48, 49]. Previous studies have found that neurons release excessive glutamate into the extracellular space of the brain's parenchyma after ischaemic stroke. A slight increase in glutamate concentration will lead to a significant increase in intracellular calcium and neuronal death [50, 51]. AST is widely distributed in all tissues, especially in the heart, liver, skeletal muscle, kidney, and even brain, while ALT is mainly found in liver tissue. This may cause AST to proliferate at a higher rate than ALT, even as the patient's condition deteriorates [52, 53]. This might be one of the reasons for the worse neurological outcomes in CA patients with a higher De-Ritis ratio in this study. According to European guidelines, neurological prognosis should not be performed before 72 hours for patients who remain comatose [54]. In view of the fact that a considerable number of CA patients with good neurological prognosis have delayed recovery, the prediction of neurological function may be even more difficult. Thus, the relationship between neurologic outcome in survivors of CA and the De-Ritis ratio needs further research.

## 5. Limitations

The preliminary findings should be viewed in terms of several limitations. First, as a retrospective, single-centre analysis with possible biases related to this design, unknown confounding factors may affect the results, regardless of the analytical adjustments. Additionally, the study design only allowed us to establish statistical associations rather than a causal relationship between the De-Ritis ratio and ICU mortality and hospital mortality. Second, we did not study the relationship between the serial changes of the De-Ritis ratio and the prognosis of CA patients. Therefore, we cannot confirm whether the De-Ritis ratio is simply the result of preexisting clinical conditions or subsequent changes caused by CA itself. Third, neuron-specific enolase (NSE), neurofilament light chain (NFL), and protein S100 beta (S100B), which were important prognostic factors, could not be evaluated by the database, although they may help to further understand the additional value of the De-Ritis ratio. Fourth, information relating to hospital infection and blood cell markers was not consistently available, which could impact the results. Finally, limited information was available as to the functional status of patients after CA.

## 6. Conclusions

An elevated De-Ritis ratio on admission was significantly associated with ICU mortality and hospital mortality after CA. It is necessary to verify the predictive value of the De-Ritis ratio after CA in multicentre trials.

## Figures and Tables

**Table 1:** Baseline clinical characteristics of the study population at CA.

Variable	All patients (*n* = 374)	Tertile 1 (*n* = 125)	Tertile 2 (*n* = 125)	Tertile 3 (*n* = 124)	*p*
Gender (men), *n* (%)	270 (72.2)	86 (68.8)	88 (70.4)	96 (77.4)	0.272
Mean age (years)	62 (51-74)	60 (51-73)	64 (53-76)	65 (51-75)	0.362
Weight (kg)	77 (67-85)	80 (67-88)	80 (66-86)	75 (68-82)	0.242
ICU length of stay (days)	4 (2-9)	4 (3-10)	5 (2-9)	4 (2-9)	0.470
APACHE II score	25 (20-29)	24 (20-29)	24 (19-29)	26 (21-30)	0.259
SOFA score	11 (9-14)	11 (9-14)	11 (9-13)	12 (9-14)	0.195
Arrest characteristics					
Bystander-witnessed CA, *n* (%)	320 (85.6)	103 (82.4)	105 (84.0)	112 (90.3)	0.171
Bystander CPR, *n* (%)	254 (67.9)	92 (73.6)	78 (62.4)	84 (67.7)	0.165
Time to ROSC (min)	15 (7-25)	14 (6-24)	15 (7-25)	15 (9-25)	0.448
Adrenaline (mg)	3 (2-5)	3 (1-5)	3 (2-6)	3 (2-5)	0.564
Out of hospital, *n* (%)	207 (55.5)	71 (56.8)	80 (64.5)	56 (45.2)	0.009
TTM, *n* (%)	331 (88.7)	111 (88.8)	112 (89.6)	108 (87.8)	0.905
Noncardiac cause, *n* (%)	153 (40.9)	46 (36.8)	48 (38.4)	59 (47.6)	0.175
Nonshockable rhythm, *n* (%)	221 (59.1)	64 (51.2)	69 (55.2)	88 (71.0)	0.004
Comorbidities					
Chronic heart failure, *n* (%)	78 (20.9)	20 (16.0)	25 (20.0)	33 (26.6)	0.115
Hypertension, *n* (%)	159 (42.5)	54 (43.2)	56 (44.8)	49 (39.5)	0.688
Coronary artery disease, *n* (%)	146 (39.0)	51 (40.8)	50 (40.0)	45 (36.3)	0.739
Diabetes, *n* (%)	91 (24.3)	24 (19.2)	31 (24.8)	36 (29.0)	0.193
COPD/asthma, *n* (%)	63 (16.8)	17 (13.6)	25 (20.0)	21 (16.9)	0.401
Neurological disease, *n* (%)	54 (14.4)	18 (14.4)	17 (13.6)	19 (15.3)	0.928
Chronic renal failure, *n* (%)	62 (16.6)	19 (15.2)	15 (12.0)	28 (22.8)	0.065
Liver cirrhosis, *n* (%)	17 (4.5)	3 (2.4)	4 (3.2)	10 (8.1)	0.068
Corticosteroids, *n* (%)	85 (22.7)	30 (24.0)	24 (19.2)	31 (25.0)	0.505
Chronic anticoagulation, *n* (%)	65 (17.4)	20 (16.0)	20 (16.0)	25 (20.2)	0.607
During ICU stay					
IABP, *n* (%)	24 (6.4)	5 (4.0)	13 (10.4)	6 (4.8)	0.081
ECMO, *n* (%)	47 (12.6)	20 (16.0)	11 (8.8)	16 (12.9)	0.227
Shock, *n* (%)	200 (53.5)	62 (49.6)	65 (52.0)	73 (58.9)	0.314
Vasopressor therapy, *n* (%)	283 (75.7)	87 (69.6)	95 (76.0)	101 (81.5)	0.093
Inotropic agents, *n* (%)	201 (53.7)	61 (48.8)	66 (52.8)	74 (59.7)	0.220
Mechanical ventilation, *n* (%)	369 (98.7)	123 (98.4)	123 (98.4)	123 (99.2)	0.820
Use of hepatotoxic drugs, *n* (%)	325 (86.9)	108 (86.4)	110 (88.0)	107 (86.3)	0.904
Paracetamol, *n* (%)	203 (54.3)	66 (52.8)	75 (60.0)	62 (50.0)	0.263
Amiodarone, *n* (%)	187 (50.0)	57 (45.6)	65 (52.0)	65 (52.4)	0.482
*β*-Lactams, *n* (%)	158 (42.2)	47 (37.6)	59 (47.2)	52 (41.9)	0.306
Quinolones, *n* (%)	7 (1.9)	0 (0.0)	3 (2.4)	4 (3.2)	0.149
Azoles, *n* (%)	8 (2.1)	4 (3.2)	2 (1.6)	2 (1.6)	0.604
Isoniazid, *n* (%)	0 (0.0)	0 (0.0)	0 (0.0)	0 (0.0)	—
TMP/SMX, *n* (%)	0 (0.0)	0 (0.0)	0 (0.0)	0 (0.0)	—
Metronidazole, *n* (%)	2 (0.5)	1 (0.8)	1 (0.8)	0 (0.0)	0.607
Chemotherapy, *n* (%)	0 (0.0)	0 (0.0)	0 (0.0)	0 (0.0)	—
CRRT, *n* (%)	61 (16.3)	15 (12.0)	21 (16.8)	25 (20.2)	0.215
HH, *n* (%)	27 (7.2)	8 (6.4)	4 (3.2)	15 (12.1)	0.023
ALF, *n* (%)	208 (55.6)	59 (47.2)	65 (52.0)	84 (67.7)	0.003
AKI, *n* (%)	221 (59.1)	68 (54.4)	75 (60.0)	78 (62.9)	0.382
Lowest platelet count (mm [3])	133 (79-187)	144 (79-194)	148 (94-193)	107 (69-169)	0.024
Lowest ScvO_2_/SvO_2_ (%)	62 (56-66)	61 (56-66)	63 (56-68)	62 (56-66)	0.662
Hematology index on admission					
Lactate (mEq l^−1^)	5.1 (4.1-7.7)	4.6 (3.8-7.2)	5.1 (4.4-7.5)	5.6 (4.2-8.3)	0.016
ScvO_2_/SvO_2_ (%)	69 (64-74)	68 (63-74)	70 (64-77)	68 (63-73)	0.050
AST (IU/L)	95 (47-193)	100 (47-193)	68 (38-134)	121 (66-242)	<0.001
ALT (IU/L)	68 (32-153)	106 (60-226)	50 (29-104)	50 (29-120)	<0.001
LDH (IU/L)	336 (240-489)	311 (225-456)	321 (235-412)	403 (262-588)	0.001
ALP (IU/L)	77 (58-106)	72 (57-95)	74 (59-97)	91 (59-137)	0.003
GGT (IU/L)	68 (42-103)	66 (40-102)	61 (36-81)	85 (55-138)	<0.001
Total bilirubin (mg dL^−1^)	0.5 (0.3-0.9)	0.5 (0.3-0.8)	0.5 (0.3-0.9)	0.6 (0.4-1.1)	0.020
APTT (sec)	32 (27-44)	31 (26-40)	32 (28-43)	35 (29-52)	0.012
PT (%)	65 (47-79)	71 (50-88)	67 (50-81)	55 (42-70)	<0.001
INR	1.3 (1.1-1.5)	1.2 (1.1-1.5)	1.2 (1.1-1.5)	1.4 (1.2-1.7)	<0.001
Platelets (mm [3])	201 (138-267)	210 (148-273)	206 (151-251)	179 (120-244)	0.044
Proteins (mg dL^−1^)	5.7 (5.0-6.3)	5.8 (5.0-6.2)	5.8 (5.0-6.6)	5.6 (5.0-6.2)	0.598
Glucose (mg dL^−1^)	200 (155-290)	215 (151-311)	214 (170-299)	180 (135-252)	0.003
pH	7.30 (7.21-7.38)	7.30 (7.22-7.38)	7.29 (7.22-7.38)	7.30 (7.19-7.38)	0.948
PaCO_2_ (mmHg)	37 (33-44)	37 (33-44)	38 (34-45)	37 (32-43)	0.303
PaO_2_ (mmHg)	111 (85-179)	114 (86-177)	111 (86-184)	108 (83-180)	0.706
MAP (mmHg)	86 (75-103)	89 (78-106)	87 (74-109)	84 (72-99)	0.040
Creatinine (mg dL^−1^)	1.2 (0.9-1.6)	1.2 (1.0-1.5)	1.2 (0.9-1.6)	1.2 (0.9-1.9)	0.699
CRP (mg dL-1)	40 (14-84)	32 (9-71)	32 (11-73)	50 (21-110)	0.008

Abbreviation: ICU: intensive care unit; CPR: cardiopulmonary resuscitation; ROSC: return of spontaneous circulation; TTM: targeted temperature management; COPD: chronic obstructive pulmonary disease; IABP: intra-aortic balloon pump; ECMO: extracorporeal membrane oxygenation; CRRT: continuous renal replacement therapy; HH: hypoxic hepatitis; ALF: acute liver failure; AKI: acute kidney injury; ScvO_2_/SvO_2_: central venous/mixed venous oxygen saturation; AST: aspartate aminotransferase; ALT: alanine aminotransferase; LDH: lactate dehydrogenase; ALP: alkaline phosphatase; GGT: *γ*-glutamyl transferase; APTT: activated partial thromboplastin time; PT: prothrombin time; INR: international normalized ratio; MAP: mean arterial pressure; CRP: C-reactive protein; APACHE: Acute Physiology and Chronic Health Evaluation; SOFA: Sequential Organ Failure Assessment.

**Table 2:** Spearman correlations analysis between De-Ritis ratio and other parameters.

Variable	*r* value	*p* value
Lowest platelet count (mm [3])	-0.117	0.024
Lactate (mEq l^−1^)	0.123	0.017
LDH (IU/L)	0.123	0.017
ALP (IU/L)	0.153	0.003
GGT (IU/L)	0.139	0.007
Total bilirubin (mg dL^−1^)	0.122	0.018
APTT	0.129	0.013
PT (%)	-0.240	<0.001
INR	0.226	<0.001
PLT (mm [3])	-0.118	0.022
Glucose (mg dL^−1^)	-0.133	0.010
CRP (mg dL^−1^)	0.159	0.002

Abbreviation: LDH: lactate dehydrogenase; ALP: alkaline phosphatase; GGT: *γ*-glutamyl transferase; APTT: activated partial thromboplastin time; PT: prothrombin time; INR: international normalized ratio; PLT: platelets; CRP: C-reactive protein.

**Table 3:** Primary and secondary outcomes.

Outcome	All patients (*n* = 374)	Tertile 1 (*n* = 125)	Tertile 2 (*n* = 125)	Tertile 3 (*n* = 124)	*p*
ICU mortality, *n* (%)	194 (51.9)	50 (40.0)	62 (49.6)	82 (66.1)	<0.001
Hospital mortality, *n* (%)	213 (57.0)	53 (42.4)	69 (55.2)	91 (73.4)	<0.001
Unfavorable neurological, *n* (%)	226 (60.4)	59 (47.2)	73 (58.4)	94 (75.8)	<0.001

Abbreviation: ICU: intensive care unit.

**Table 4:** Odds ratios (95% confidence intervals) for ICU mortality, hospital death, and unfavorable neurological outcome by De-Ritis level tertiles.

Outcome	Crude OR (95% CI)	*p*	Adjusted OR (95% CI)	*p*
ICU mortality				
De-Ritis tertiles				
Tertile 1	Reference		Reference	
Tertile 2	1.476 (0.894-2.436)	0.128	1.283 (0.703-2.341)	0.418
Tertile 3	2.929 (1.748-4.907)	<0.001	2.691 (1.443-5.018)	0.002
Hospital mortality				
De-Ritis tertiles				
Tertile 1	Reference		Reference	
Tertile 2	1.674 (1.015-2.760)	0.044	1.739 (0.952-3.180)	0.072
Tertile 3	3.746 (2.198-6.386)	<0.001	3.243 (1.716-6.132)	<0.001
Unfavorable neurological outcome				
De-Ritis tertiles				
Tertile 1	Reference		Reference	
Tertile 2	1.570 (0.953-2.589)	0.077	1.403 (0.772-2.550)	0.267
Tertile 3	3.505 (2.041-6.018)	<0.001	2.904 (1.532-5.505)	0.001

Abbreviation: ICU: intensive care unit.

**Table 5:** Unadjusted and adjusted effects on ICU mortality, hospital death and unfavorable neurological outcome.

Outcome	Variable	Crude OR (95% CI)	*p*	Adjusted OR (95% CI)	*p*
ICU mortality	De-Ritis ratio	1.450 (1.127-1.865)	0.004	1.455 (1.088-1.946)	0.011
	AST	1.001 (1.000-1.002)	0.060	1.000 (0.999-1.001)	0.545
	ALT	1.001 (1.000-1.002)	0.085	1.000 (0.999-1.001)	0.790
Hospital mortality	De-Ritis ratio	1.425 (1.100-1.846)	0.007	1.378 (1.031-1.842)	0.030
	AST	1.001 (1.000-1.001)	0.194	1.000 (1.000-1.001)	0.835
	ALT	1.001 (1.000-1.001)	0.225	1.000 (0.999-1.001)	0.756
Unfavorable neurological outcome	De-Ritis ratio	1.362 (1.051-1.765)	0.020	1.281 (0.940-1.745)	0.117
	AST	1.001 (1.000-1.001)	0.146	1.000 (0.999-1.001)	0.706
	ALT	1.001 (1.000-1.002)	0.200	1.000 (0.999-1.001)	0.999

Abbreviation: ICU: intensive care unit; AST: aspartate aminotransferase; ALT: alanine aminotransferase.

**Table 6:** Comparison of different prognostic models on CA patients.

Model predictors	Likelihood ratio test *χ*^2^	AIC	*p* value	AUROC
ICU mortality				
Model 1	53.58	476.37	<0.001	0.715
Model 2	68.45	463.50	<0.001	0.738
Hospital mortality				
Model 1	52.41	470.81	<0.001	0.714
Model 2	74.86	450.36	<0.001	0.752
Unfavorable neurological outcome				
Model 1	45.26	468.82	<0.001	0.700
Model 2	64.96	451.12	<0.001	0.736

Abbreviation: AIC: Akaike information criterion; AUROC: area under the receiver operating characteristics curve. Model 1 included age, sex, adrenaline, bystander CPR (yes/no), and serum lactate level at admission. Model 2 included model 1 plus the De-Ritis ratio.

## Data Availability

The data set is available on Dryad via: 10.5061/dryad.qv6fp83.

## Abbreviations

CA: Cardiac arrest abbreviation
CPR: Cardiopulmonary resuscitation
ROSC: Restoration of spontaneous circulation
AST: Aspartate aminotransferase
ALT: Alanine aminotransferase
CVD: Cardiovascular disease
CHF: Chronic heart failure
CAD: Coronary heart disease
AMI: Acute myocardial infarction
COPD: Chronic obstructive pulmonary disease
GCS: Glasgow Coma Scale
IHCA: In-hospital CA
OHCA: Out-hospital CA
TTM: Targeted temperature management
SOFA: Sequential Organ Failure Assessment
APACHE: Acute Physiology and Chronic Health Evaluation
LDH: Lactate dehydrogenase
PT: Prothrombin time
INR: International normalized ratio
LDH: Lactate dehydrogenase
GGT: *γ*-Glutamyl transferase
CRRT: Continuous renal replacement therapy
IABP: Intra-aortic balloon pump
ECMO: Extracorporeal membrane oxygenation
CHF: Chronic heart failure
HTN or HT: Hypertension
CRF: Chronic renal failure
SBP: Systolic blood pressure
DBP: Diastolic blood pressure
DM: Diabetes mellitus
ALF: Acute liver failure
INR: International normalized ratio
CLD: Chronic liver disease
HH: Hypoxic hepatitis
AKI: Acute kidney injury
ICU: Intensive care unit
CPC: Cerebral performance categories
ScvO_2_/SvO_2_: Central venous/mixed venous oxygen saturation
ALP: Alkaline phosphatase
APTT: Activated partial thromboplastin time
MAP: Mean arterial pressure
CRP: C-reactive protein
ROC: Receiver operator curves
AUC: Area under the curve.
